# Enhancing network lifetime in WSNs through coot algorithm-based energy management model

**DOI:** 10.1016/j.mex.2025.103176

**Published:** 2025-01-16

**Authors:** Namita Shinde, Dr. Vinod H․ Patil

**Affiliations:** Bharati Vidyapeeth (Deemed to be University), College of Engineering, Pune-Satara Road, Pune, 411043

**Keywords:** Swarm intelligence, Wireless sensor network (WSN), Cluster head selection, Energy efficient routing, Coot optimization and network lifecycle, Enhancing Network Lifetime in WSNs through Coot Algorithm-Based Energy Management Model

## Abstract

To improve the performance of Wireless Sensor Networks (WSN), this study offers a novel energy-efficient clustering and routing technique based on the Coot Optimization Algorithm (COA). This addresses issues such as high energy consumption, communication delays, and security.

To ensure energy savings and network reliability, the fitness function evaluates cluster heads and best routes based on constraints.

COOT outperforms other Metaheuristics Algorithms like Butterfly Optimization Algorithm, Genetic Algorithm, Tunicate Swarm Gray Wolf Optimization Algorithm, and Bird Swarm Algorithm in simulation with performance measurements and enhancing network functionality and protection.

Key methodology points include:

• Proposed a multiple constraints clustering and routing technique using COAto solve the most crucial issues that arise in WSNs.
• Integrated an advanced fitness function that determines cluster head selection, and the routing path based on residual energy, delay, security, trust, distance, and link quality so that energy load is evenly distributed and credible data flow is maintained across the network and made Innovative and Effective Solution.
• Proven Results Demonstrated superior network performance, achieving the lowest delay, highest network lifetime (3571 rounds) and enhanced security (0.8) and trust (0.6) compared to existing algorithms with less energy consumption, making it the most suitable solution for WSN performance improvement.

## Graphical abstract

**Figure:**
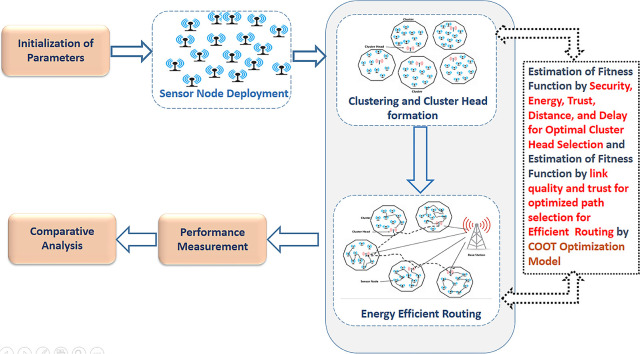

## Nomenclature

***Abbreviation***: ***Description***
*COA*: COOT Optiization Algorithm
*BFO*: Bacterial Foraging Optimization
*BOA*: Butterfly Optimization Algorithm
*BSA*: Bird Swarm Algorithm
*CH*: Cluster Head
*BS*: Base Station
*GA*: Genetic Algorithm
*IoT*: Internet of Things
*SI*: Swarm Intelligence
*TSGWO*: Tunicate Swarm Grey Wolf Optimization
*WSN*: Wireless Sensor Network
*PDR*: Packet Delivery Ratio
*TCSC*: Thyristor Controlled Series Capacitor
*PVG*: Photovoltaic Generators
*RDN*: Radial Distribution Networks
*VANET*: Vehicular Ad-hoc Network

Specifications table

**Table:** 

Subject area:	Computer Sciences
More specific subject area:	Wireless Sensor Network
Name of your method:	Enhancing Network Lifetime in WSNs through Coot Algorithm-Based Energy Management Model
Name and reference of the original method:	*COOT Algorithm* *Naruei, I., & Keynia, F. (2021). A New Optimization Method Based on COOT Bird Natural Life Model. Expert Syst. Appl., 183, 115,352.*Https://Doi.Org/10.1016/J.ESWA.2021.115352
Resource availability:	—

### Background

**Fig. 1:**
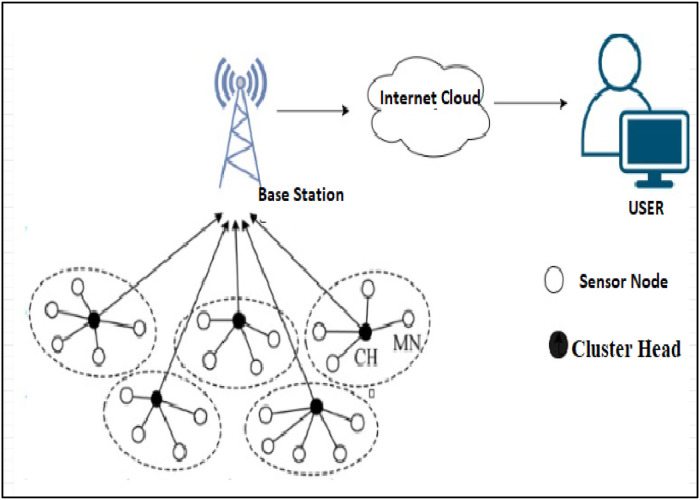
Basic Structure of Wireless sensor Network.

Power management is an enormous challenge in WSNs,assensor nodes are equipped with constrained energy resources. Basic structure of WSN is as shown in Fig. 1. Proper control of the energy is required to prolong the lifetime of the network, guarantee reliable communication, and provide services that need reliability and data delivery with low delays [1]. Conventional methods of using algorithms such as LEACH (Low Energy Adaptive Clustering Hierarchy) [2] and PEGASIS (Power-Efficient Gathering in Sensor Information Systems) [3] basically focuses on energy conservation mainly through clustering or routing methods. However, these deterministic approaches are far from being effective in a dynamic network in which the nodes’ energy levels are not constant while the communication patterns are not regular [4,5]. Furthermore, deterministic algorithms like LEACH proposed to minimize energy expenses again by randomly rotating the Cluster Heads (CH's) irredeemably, again it does not consider real-time factors such as the distance between CHs and the Base Station (BS) etc. This results in better than optimal CH selection, high power consumption and consequently less longevity of the network. Likewise, other stochastic approaches like PSO and GA have been applied for routing and clustering though these methods suffer from few drawbacks like slow convergence rate, scalability issues, and poor energy consideration [6].

To overcome these limitations, the metaheuristic optimization algorithms such as the COOT Optimization Algorithm (COA) can be applied because it is flexible and dynamic in nature. Metaheuristics enable CH selection along with energy sensitive routing and path variability owing to the real time Status of nodes and the distance between them. This is important in order to avoid early disconnecting of some nodes in the network and to maintain equal energy and load. Contribution of this Paper includes following points:

- CH selection using an optimization of distance-based function to find CH closer to the base station so that power consumption is minimized.
- Minimizing transmission delays by selecting the most appropriate communication channels, enhancing the speed and efficiency of data transfer across the network.
- The COOT algorithm provides a more robust and efficient solution for WSNs, resulting in enhanced network performance, with secured transmission and extended network lifetime.That has been proved by comparing with existing traditional methods.

This contribution is a breakthrough over the previous approaches since it identifies and fills the key knowledge gaps concerning energy efficiency, network durability, and data transfer rates. The traditional work in the field of clustering and routing has not been effectively solved by deterministic as well as existing non-deterministic algorithms. However, the work presented by the COOT algorithm is entirely new. Coming up with an adaptive decision criterion that hinders the normal changes in the real network that the WSNs portray it brings a new and efficient solution to energy optimization problems.

This paper is organized as follows: Section 2 covers the related work as well as the shortcomings of prior strategies. Section 3 puts forward the proposed methodology and Section 4 describes the simulation and findings of the study. Finally, Section 5 is devoted to the conclusion of the paper and the indications towards the future research.

#### Related work

WSNs energy optimization has received substantial attention and enhanced through several metaheuristic algorithms. So, these algorithms, derived from natural systems They can provide unstructured approaches for the given constant change in the network environment. The following section addresses the most engaging algorithms that have been developed and demonstrates COOT optimization algorithm's contribution and shortcomings in relation to them.

One such metaheuristic technique is the Butterfly Optimization Algorithm (BOA), which mimics the foraging behavior of butterflies [7,8]. It has been successfully used for WSNs for activities such as node positioning and energy efficiency [9], outperforming conventional algorithms in a range of areas including engineering and Unmanned Aerial Vehicle (UAV) trajectory planning [10]. Because of its generic nature and capacity to learn, the usage of BOA is especially beneficial when operating in considered transient environments in order to preserve the performance of network and the resources of WSN [11]. Another important algorithm is the Bird Swarm Algorithm (BSA) that emulates the process of how birds forage or search for food react on threatsor fly [12],. This is evident with BSA and other versions such as the Improved Bird Swarm Algorithm (IBSA) and the Multi-objective Bird Swarm Algorithm (MBSA) [[13], [14], [15]], by proving to some extent the efficacy of Bird Swarm Algorithms on optimization problems. These algorithms are very useful in improving the energy use and longevity of WSNs from the two facets of optimization- the exploration and the exploitation. BOA and BSA are very pertinent contributions to nature-inspired optimization methods. It provides reliable solutions to the energy and routing problems in WSNs. The Genetic Algorithm (GA) in WSNs helps to choose the best number of clusters [16], the best cluster head and designated the best path for the routing system so as to maintain energy utilization and hence the life span of the network [17,18].

It aims at reproducing and adapting certain patterns drawn from the real world of natural selection for meter placement, coverage, and clustering. Multi-Objective Deployment Strategy (MODS) [19], and similar evolutionary computational methods such Non-dominated Sorting Genetic Algorithm II (NSGA-II) have shown how GA can be used in optimizing the deployment of WSN [20]. GA is especially perfect for solving large scale problems and can be easily adapted for a variety of problems, thereby making it a very effective means of increasing overall benefits of WSN. The Tunicate Swarm Grey Wolf Optimization (TSGWO) involves the social characteristics of tunicates and grey wolves to solve challenging problems, as resource utilization and routing in WSNs [21].Fractional Gravitational Search Algorithm (FGSA) for selection of cluster head [22] and employs TSGWO for multipath Routing taking in to consideration QoS aspects such as delay, energy, and link lifetime. TSGWO has been compared with a number of problems and it was found that TSGWO is very efficient in optimizing function, sensitive, and scalable [23].

The discussed algorithms have several disadvantages when dealing with WSN optimization issue, mainly the convergence rate, the energy consumption, and the computational cost. GA does perform well; however, the problem with the proposed approach is that it takes a long time for the algorithm to converge and is computationally heavy. It is not very useful for energy-efficient solutions. While the BOA can be used for node positioning and for minimizing the energy consumption, it gets easily stuck in the local optimum and therefore the energy consumption is not optimal. It is also found that the local optimum trap problem and energy utilization balance between exploration and exploitation can be issues of BSA. Last of all, the TSGWO has shown enhanced resource utilization, but has shown limitations in scalability wherein it is less energy efficient in large networks. Such restrictions suggest that there is a dire need to develop energy efficient algorithms such as the COOT optimization algorithm.

The Coot Optimization Algorithm is intended to overcome the shortcomings of other metaheuristic techniques by including chaotic systems and Lévy flights. These features facilitate exploration and exploitation of COOT optimization algorithm's thus enabling the context to enjoy better convergence rates and flexibility. Thus COA successfully addressed the problems indicted by GA, BOA BSA and TSGWO such as slow convergence, local optima and scalability. As can be concluded from the empirical results, the COA scheme outperforms others in energy consumption and network lifetime in dynamic and large-scale scenarios. The enhanced techniques involved in algorithm give it more strength of being used as a solution for improving WSNs where conventional models fails. Altogether, though GA, BOA, BSA and TSGWO have made great advancement in the optimization. Each of them has some pros and cons that affect their suitability to dynamic or large scale WSN networks details shared in Table 1. COA helps to overcome these limitations using the modern techniques and enables the improvement of energy consumption rate, time convergence, and flexibility, which makes its application valuable for enhancing the field of WSN optimization.

**Table 1:** Comparing Optimization Algorithms for Routing and Clustering in WSN and the Gaps Covered by COA.

Algorithm	Key Features	Advantages	Limitations	Gaps Addressed by COA
Genetic Algorithm (GA)	Evolutionary algorithm for clustering and routing [16-18]	Effective for large-scale problems; multi-objective optimization	Slow convergence; high computational cost; parameter sensitivity	Faster convergence; reduced computational cost with chaotic systems
Butterfly Optimization Algorithm (BOA)	Mimics butterfly foraging behavior [9,10]	Efficient in static WSN environments; adaptable to node positioning	Local optima issues in dynamic environments; energy inefficiency	Improved exploration via chaotic systems to avoid local optima
Bird Swarm Algorithm (BSA)	Simulates bird foraging and flocking [12,13]	Good balance between exploration and exploitation	Limited adaptability in dynamic networks	Better adaptability in dynamic conditions with Lévy flights
Tunicate Swarm Grey Wolf Optimization (TSGWO)	Combines social behaviors of tunicates and grey wolves [21,22]	Efficient in multipath routing and resource optimization	Scalability issues in large, complex networks; energy inefficiency	Enhanced scalability and energy efficiency with COA's robust approach

#### Research work on coot optimization algorithm

The major enhancements are as follows: firstly, the employment of chaotic systems to describe population initialization; secondly, Lévy flights for the perturbation processes; finally, oppositional-based learning in order to improve the operations of the local search [24,25]. These optimizations extend the applicability of the algorithm. Allows it to better consider and relocate to the best solution space. They also prevent the situation of getting fixed or stuck at a local optima; hence, they are likely to offer better accuracy of solutions within less time when compared to traditional algorithmic techniques used in solving the same problems. [26,27]. COOT's efficiency is further substantiated in other uses, for instance, coverage optimization in WSN where the algorithm leverages on the network rate and energy levels in enhancing network performance as compared to other algorithms [28]. Shuffled Shepherd-Coot (SS-Coot), a variant with herding behavior, improves its optimization performance in tasks such as privacy preservation of a source location [29]. COOT has also been used in other power systems optimization such as optimization of Thyristor controlled series capacitor optimization and position, and photovoltaic generator placement to minimize energy losses and improve power quality [30]. Also, extension of COOT based hybrid models including Energy Aware Multi-Hop Routing Protocol (ICOOT-EAMHRP) for Vehicular Ad-hoc Network (VANET) and (COOT bird natural life model- Artificial Neural Network (COOT-ANN)) for Meteorological Prediction shows that the algorithm can be applied to different fields [[31], [32]-33]. In conclusion, scalability, energy conservation and adaptability to environment dynamics makes COOT to be advantageous for real-world WSN application.

### Method details

This Study presents a clustering and routing techniques for fully energy-efficient Wireless Sensor Networks (WSNs) based on the COOT optimization algorithm. The focus is on energy efficiency, network security in the context of Internet of Things, delay reduction and distance shortening for trans-nodes communication, trustworthiness. To derive balanced clusters, cluster head (CH) selection is an essential parameter in a clustering process. The proposed clustering method selects CHs based on energy efficiency, security, trust, delay, and distance.

**Fig. 2:**
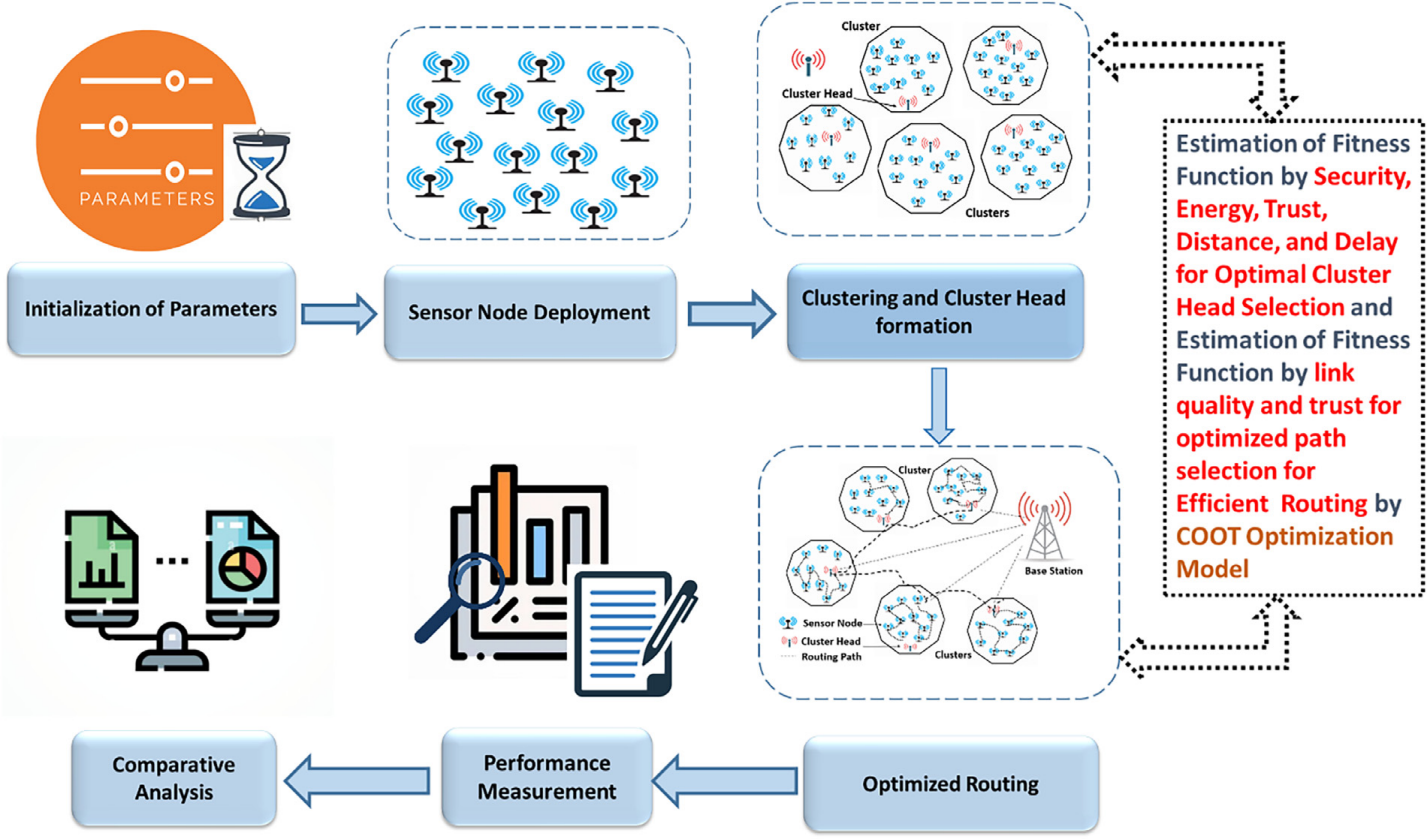
Block Diagram of Proposed Methodology.

In routing phase, a route is searched based on trust and link quality that leads to minimum energy dissipation for successful data transmission. The work combines the COOT optimization algorithm for clustering with that for routing and utilize its exploration and exploitation to improve network performance through adapting node positions dynamically and choosing the best optimal routes. So, the detailed methodology consisting of steps initialization, clustering, routing, and optimization phases, alongside mathematical formulations for each process are explained here. Fig. 2 shows block diagram of the proposed methodology.

#### System and network model

Model can be defined as a WSN that contains N mobile sensor nodes that are randomly deployed over a specific area. Each node i is defined by the tuple$\left(x_{i},y_{i},E_{i},T_{i},S_{i}\right)$, where:

• $\left(x_{i},y_{i}\right)$ are the coordinates of i node,
• $E_{i}$ is the level of energy,
• $T_{i}$ is the trust level,
• $S_{i}$ is the security level

Nodes communicate to each other through multi-hop transmission and each node has transmission range R​WSN works in rounds, with CHs being first selected and then routing is performed from the nodes to a BS where data is relayed.

I. Clustering Phase

**Fig. 3:**
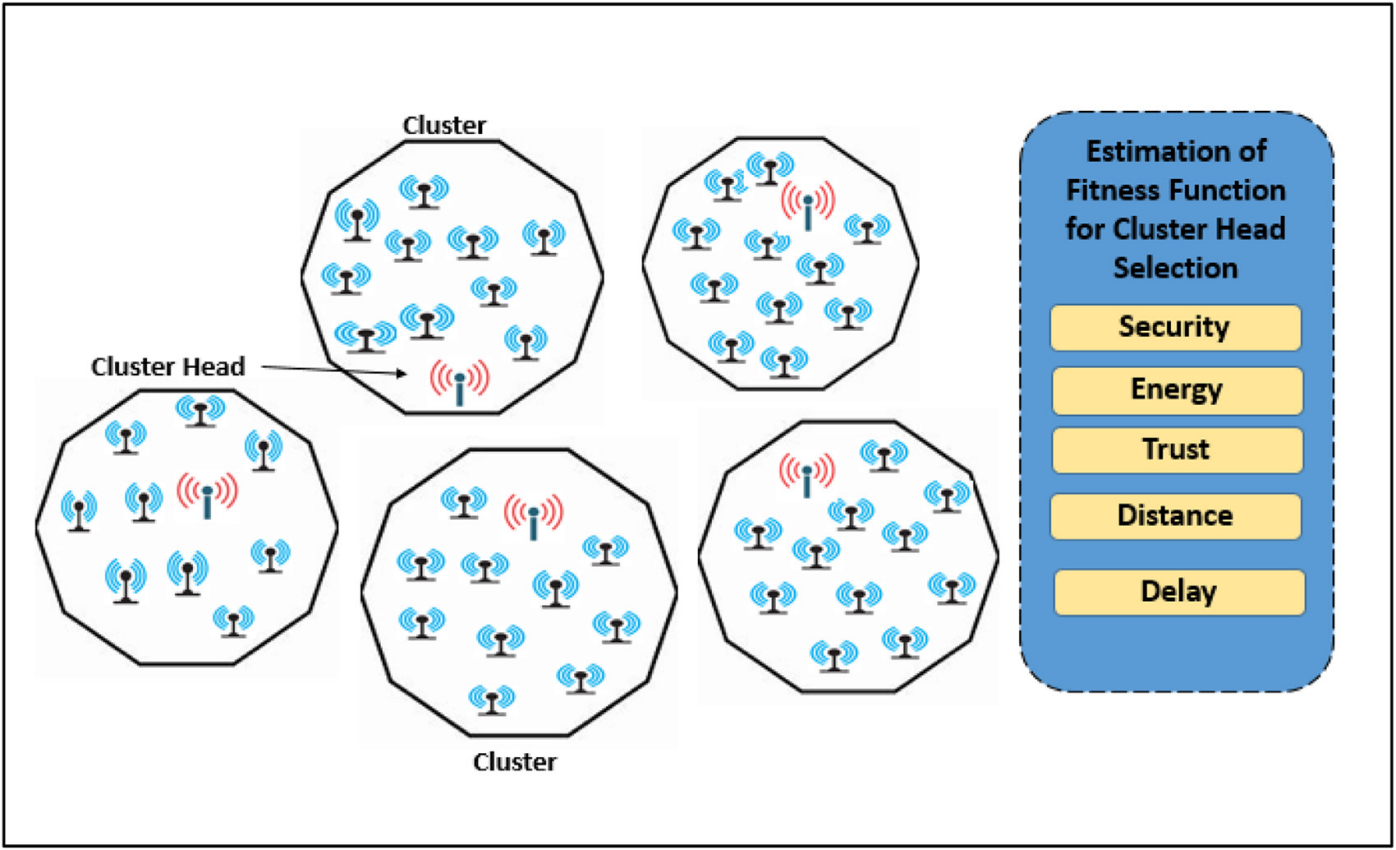
Architecture for CH selection by COOT optimization Model.

Cluster head (CH) selection is a crucial clustering process parameter that determines the creation of balanced clusters. The energy efficiency, security, trust, latency, and distance constraints are considered in calculation fitness function by the suggested clustering algorithm to choose CHs shown in Fig. 3.

Step 1 Node Initialization

At this point, all nodes initialize their own local state by loading all the parameters and evaluating the network topology through this neighbor discovery mechanism. All the nodes in the network are initialized with some parameter values; for instance, such as energy levels, trust, security, etc. Then these parameters are communicated by every neighbor node to create a perfect outlook of the network.

In the initialization process of WSN, Nodes are randomly spread over the given area. If the deployment area is a 100m×100m grid, each node's coordinates $\left(x_{i},y_{i}\right)$ could be: $x_{i}\in \left[0,100\right],\;y_{i}\in \left[0,100\right]$.

The coordinate settings are assigned random values within the network dimensions for uniform distribution. (xi​,yi​).

Each node is assigned with an initial fixed amount of energy $E_{i}\;$.Which is typically set at 1 Joule, this is the available energy for communication and computational work. Trust values are generally normalized between 0 and 1, where 1 represents full trust. Typically, it would be $T_{i}\in \left[0.5,1.0\right]$ meaning at the initial state, an assumption is made that nodes are reliable. Like trust, security can be normalized between 0 and 1, with 1 representing high security.$S_{i}\in \left[0.6,1.0\right]$. Nodes may be initialized with different security levels, depending on their capacity for encryption or resistance to attacks. For every node, transmission range, usually $R_{t}=20\;\text{meters},$ shows how far nodes can communicate with each other. All these parameters put the network in its basic state before the clustering and routing phases. Following are the values initialized before the simulation begins given in Table 2.

**Table 2:** Initialization of Parameters.

Parameter	Description	Values to be Initialized
$x_{i},y_{i}$	Coordinates of node i in the deployment area	Randomly generated within the deployment area (e.g. 0 to 1000 m)
$E_{i}$	Initial energy level of node i	2 J (full energy)
$T_{i}$	Initial trust level of node i	0.5 (initial trustworthiness)
$S_{i}$	Security level of node i	0.8 (initial security level)
$R_{t}$	Transmission range of every node i	100 m
N	Number of sensor nodes in the network	100 nodes
Rounds	Number of rounds for network operation	1000 rounds
$P_{CH}$	Probability of a node becoming a cluster head	0.05
$E_{threshold}$	Energy threshold for CH selection	0.5 J
BS	Coordinates of the Base Station	Fixed at (500, 500) (center of deployment area)
$d_{i,CH}$	Distance from node i to its cluster head	Computed during the clustering phase

Step 2 Fitness Function for CH Selection

Each node computes a fitness value based on five constraints: Security, Energy, Trust, Distance, and Delay. The weights w1, w2, w3, w4, w5 are chosen based on the importance of each constraint. The fitness function $F_{i}$ of node i is defined as shown in Eq. (1):

(1) $$F_{i}=w_{1}\cdot S_{i}+w_{2}\cdot E_{i}+w_{3}\cdot T_{i}+w_{4}\cdot \left(\frac{1}{D_{i}}\right)+w_{5}\cdot \left(\frac{1}{L_{i}}\right)$$

Where:

• $S_{i}$ is the node's security level.
• $E_{i}$ is the node's energy level, normalized by initial energy $E_{\text{initial}\;}$.
• $T_{i}$ is the node's trust score, which is a weighted combination of direct and indirect trust.
• $D_{i}$ is the Euclidean distance between the node and its neighboring nodes or CH.
• $L_{i}$ is the delay in communication (distance over transmission speed).

i. Security Constraint

• Security constraint *S_i_* evaluates the level of security in node i. Nodes are assigned security values based on some risk assessment. Nodes in secure mode attain higher security values in between 0.8 and 1. Nodes in the dangerous mode have the security values in the range from 0.5 to 0.8. All nodes in a high-risk mode are assigned security values below 0.5. For the clustering process, the final security score for node i is given by Eq. (2):
  (2) $$S_{i}=p_{s}\cdot S_{\text{secure}\;}+p_{r}\cdot S_{\text{risky}\;}+p_{hr}\cdot S_{\text{high}-\text{risk}\;}$$
• Where $p_{s},p_{r},p_{hr}$ are the probabilities of nodes operating in secure, risky, and high-risk modes respectively, and $p_{s}+p_{r}+p_{hr}=1$.

ii. Energy Constraint

Energy *E_i_* is an important factor, considering whether a node should act as a CH or not. The residual energy of node i is normalized with the initial energy level at which it was deployed given by Eq. (3).

(3) $$Ei=\frac{E_{\text{current}\;}}{E_{\text{initial}\;}}$$

Where:

• $E_{\text{current}\;}$ is the current energy level of node i,
• $E_{\text{initial}\;}$ is the initial energy level of node i.

Higher energy levels Nodes are prioritized to become CHs.

iii. Trust Constraint

*T_i_* is computed as a function of direct and indirect trust components. The direct trust value T ``direct” is a function of the history of interactions of the node, while the indirect trust T ``indirect” is a function of recommendations from neighboring nodes. The trust value for the node i is given by Eq. (4):

(4) $$T_{i}=\alpha \cdot T_{\text{direct}\;}+\left(1-\alpha \right)\cdot T_{\text{indirect}\;}$$

Where α is a weight (e.g. 0.5) assigned to direct trust.

• Direct Trust:
  (5) $$T_{\text{direct}\;}=\frac{E_{i}}{D_{i}}$$

Where $D_{i}$ is the distance between node i and its CH.

• Indirect Trust:
  (6) $$T_{\text{indirect}\;}=\sum_{j=1}^{N}T_{ij}$$

Where $T_{ij}$ is the trust score assigned to node i by nodej.

iv. Distance Constraint

Moreover, communication energy consumption is dependent on the distance between a node and its CH. The Euclidean distance Di between node i and the base station or neighboring nodes is defined as in

(7) $$D_{i}=\sqrt{{\left(x_{\text{BS}}-x_{i}\right)}^{2}+{\left(y_{\text{BS}}-y_{i}\right)}^{2}}$$

Where $\left(x_{\text{BS}},y_{\text{BS}}\right)$the coordinates of the base are station, and $\left(x_{i},y_{i}\right)$ are the coordinates of nodei. Nodes closer to the base station or other CHs are favored in the CH selection process.

v. Delay Constraint

It depends on the distance divided by the transmission speedv. For node i, $L_{i}$is given by the following for nodei, the delay is calculated as in Eq. (8):

(8) $$L_{i}=\frac{D_{i}}{v}$$

Where $D_{i}$ is the distance from node i to its CH or base station, and v is the transmission speed. The delay should be minimized for efficient communication to happen, which proves to be critical in dynamic MANET environments.

The node having the highest fitness score is selected as the CH. This multi-constraint optimization procedure does ensure that the selected CH would maximize security, energy efficiency, and trust, minimize communication delays, and reduce transmission energy consumption. The use of these detailed mathematical formulations ensures an energy-efficient and secure clustering mechanism, and thereby makes the network perform better in MANET environments.

Step 3 CH Selection Using COOT Optimization

The COOT algorithm selects CHs by maximizing the fitness function of nodes. The population of candidate CHs is updated iteratively as follows as in Eq. (9):

(9) $$P_{i}\left(t+1\right)=P_{i}\left(t\right)+\beta \cdot \left(P_{j}\left(t\right)-P_{i}\left(t\right)\right)+\gamma \cdot R$$

Where:

• $\;P_{i}\left(t\right)$ is the position (fitness) of node i at iteration t.
• $P_{j}\left(t\right)$ is the position of a neighboring node.
• β is a factor controlling exploration.
• γ introduces randomness for exploration.
• R is a random value ensuring diversity.

Nodes with the highest fitness values are selected as CHs.

Step 4 Exporting data to CH

The CH waits for each member node to send its measured data. Once all data have been received, the CH aggregates it together and readies itself for the return transmission toward the Base Station (BS).

II. Routing Phase

**Fig. 4:**
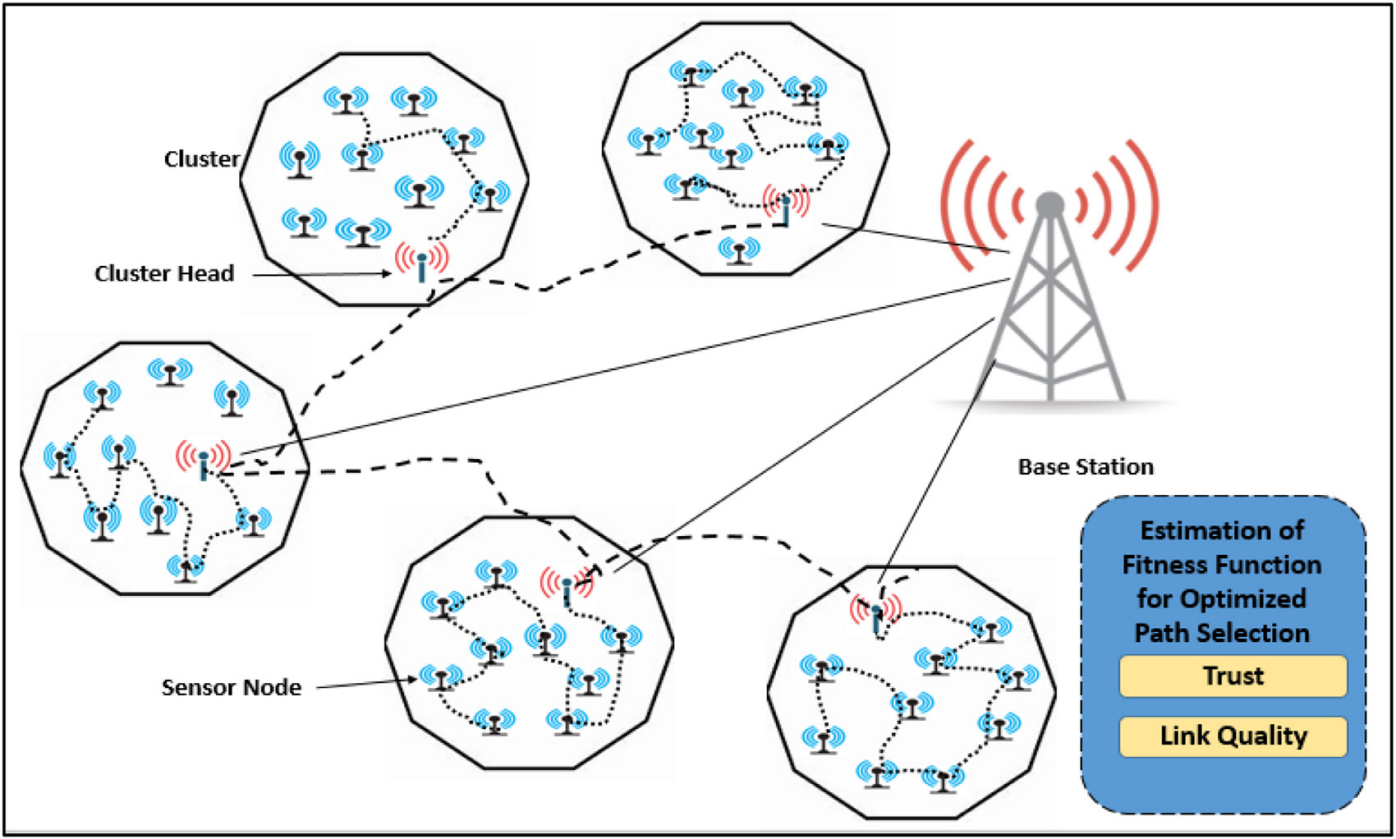
Architecture for Optimal Routing by COOT Optimization Model.

During the routing process, the selected optimal CHs are executed by using the COOT optimization algorithm for attaining optimal paths from CHs to BS with minimal energy consumption and maximum data reliability. The routing process considers the parameters of link quality and trust to ensure secure efficient data transmission shown in Fig. 4.

Step 1 population initialization

In this step, we initialize a population of routes. A route is a sequence of intermediate nodes in an order from the source node to the destination node. We generate the initial population as randomly and represents different routing paths in the MANET Mathematically,

$$R=\left\{R_{1},R_{2},\ldots ,R_{n}\right\}$$

Where:

• R is the set of possible routing paths.
• $R_{i}$ is a path containing nodes $N_{1},N_{2},\ldots ,N_{k}$ from source to destination.

Step 2 Optimization Experimentation stage

In this step, the optimization algorithm's COOT exploration mechanism is utilized to identify neighbors with better routing paths. Exploration occurs based on the computed fitness values of previous steps, which include energy consumption, link quality, and reliability.

Step 3 Exploration:

In this phase, the COOT algorithm will use a random exploration strategy. Each node will explore some of the neighboring nodes to find better routes. It can be formulated mathematically as a search space problem: exploration. Mathematically, the search behavior of the nodes can be represented as Eq. (10):

(10) $$P_{i}^{\text{new}\;}=P_{i}^{\text{current}\;}+r_{1}\cdot \left(X_{j}^{\text{best}\;}-P_{i}^{\text{current}\;}\right)$$

Where:

• $P_{i}^{\text{new}\;}$ is the new position (routing path) of node i after exploration.
• $P_{i}^{\text{current}\;}$ is the current position (routing path) of node i.
• $X_{j}^{best}$ is the best-known position (routing path) based on the neighboring nodes.
• $r_{1}$ is a random value in the range [0,1] that introduces stochasticity into the exploration process.

This formula ensures that all the nodes explore the neighboring solution space based on the best-known routes while allowing some randomness to escape from local optima.

Step 4 Fitness Update:

After exploration, the node will update its fitness values according to new routes discovered. The fitness function reevaluate is presented as follows Eq. (11):

(11) $$F\left(i\right)=w_{E}\cdot \frac{1}{E\left(i\right)}+w_{T}\cdot T\left(i\right)+w_{LQ}\cdot LQ\left(i\right)$$

Where:

• E(i) is the energy consumed by the node.
• T(i) is the trust value of the node.
• LQ(i) is the link quality of the node.
• $w_{E},w_{T}$, and $w_{LQ}$ are the weights assigned to energy consumption, trust, and link quality, respectively.

There are Link Quality (LQ) and Trust (T) which play an essential role in deciding the fitness of a pathway. This approach ensures the selection of extremely reliable, low-power and secure connections for both clustering and routing. Link Quality measures the reliability of communication between any two nodes. That includes packet loss, interference and the strength of the signal. A reliable link provides low packet loss and minimal fluctuation in communication, which is crucial for data forwarding over the WSN.

Two nodes' Link Quality (LQ) is given by Eq. (12)

(12) $$LQ\left(i,j\right)=\frac{\text{SNR}\left(i,j\right)}{\text{PDR}\left(i,j\right)}$$

Where:

• SNR(i,j) Shows the Signal-to-Noise Ratio between nodes i andj. It indicates the clarity of the communication signal relative to background noise.
• PDR(i,j) is the Packet Delivery Ratio, calculated as shown in Eq. (13):
  (13) $$\text{PDR}\left(i,j\right)=\frac{N_{pr}\left(i,j\right)}{N_{pt}\left(i,j\right)}$$

Where:

• $\;N_{pr}\left(i,j\right)$ is the number of packets received by node j from node i.
• $\;N_{pt}\left(i,j\right)$ is the number of packets transmitted from node i to node j.

A high value of LQ(i,j) indicates a high-quality, reliable link with minimal packet loss and good signal strength.

In the case of the failure of a path due to node mobility, energy depletion, or decline in Trust or Link Quality: The COOT optimization algorithm keeps re-evaluating the available paths dynamically with the help of updated fitness values. Alternative routes are chosen and high-fitness nodes are preferred so as not to affect the reliability and security of the data transmission. The fitness function, which includes LQ and Trust, can select Cluster Heads and the routing path in WSNs robustly and securely. With different weights of parameters, the network can achieve a higher reliability, security, energy performance in dynamic and resource constrained environment.

Step 5 Chain Movement (Cooperation among Nodes)

In the chain movement phase, the nodes update all information about the neighboring nodes' positions, energy levels, trust, and the quality of links and share it with each other. These nodes can learn new routes based on cooperation with better paths available in their neighbors' vicinity. Chain motion can be simulated by updating the position of node ni following the performance of its neighboring nodes nj. The motion equation is defined as Eq. (14):

(14) $$P_{i}^{\text{new}\;}=P_{i}^{\text{current}\;}+r_{2}\cdot \left(P_{j}^{\text{current}\;}-P_{i}^{\text{current}\;}\right)+r_{3}\cdot \left(LQ_{j}-LQ_{i}\right)+r_{4}\cdot \left(T_{j}-T_{i}\right)$$

Where:

• $P_{i}^{\text{new}\;}$ is the updated routing path for node i.
• $P_{j}^{\text{current}\;}$ is the current position of a neighboring node j.
• $LQ_{j}$ and $LQ_{i}$ are the link qualities of nodes j and i, respectively.
• $T_{j}$ and $T_{i}$ are the trust values of nodes j and i, respectively.
• $r_{2},r_{3}$, and $r_{4}$ are random values between [0,1] that govern This equation ensures that nodes converge toward more reliable routes, which have higher values of trust and better link quality.

Step 6 Chain Movement Fitness Calculation:

At this point in time, each node compares its fitness after chain movement and makes the necessary modifications in its path.

Step 7 Leader Movement (Global Search)

The node having the highest fitness value is regarded as a leader in the movement phase of the leader. In this phase, the leader's help the other nodes move toward better routing solutions. Leader movement is that part of the global search of the COOT algorithm because of which the best nodes decide the search direction towards more promising areas of solution space. All other nodes would be led by broadcasting leader nodes as they know best from where they are. The equation that governs the movement of leaders is as follows Eq. (15)

(15) $$P_{i}^{\text{new}\;}=P_{i}^{\text{current}\;}+\gamma \cdot \left(P_{\text{leader}\;}^{\text{best}\;}-P_{i}^{\text{current}\;}\right)$$

Where:

• $P_{i}^{\text{new}\;}$ is the new position (routing path) of node i.
• $P_{\text{leader}\;}^{\text{best}\;}$ is the position of the leader node with the best fitness.
• γ is a control parameter that dictates how strongly the node follows the leader.

Leader movement allows the algorithm to converge more rapidly by exploiting the best-known solutions.

#### Global search fitness update

After each iteration of a movement of leaders, fitness for all nodes is updated again. In the sense of leader's movement, all nodes update their routes, along with their corresponding fitness values.

Step 8 Termination and Selection of Optimal Route

**Fig. 5:**
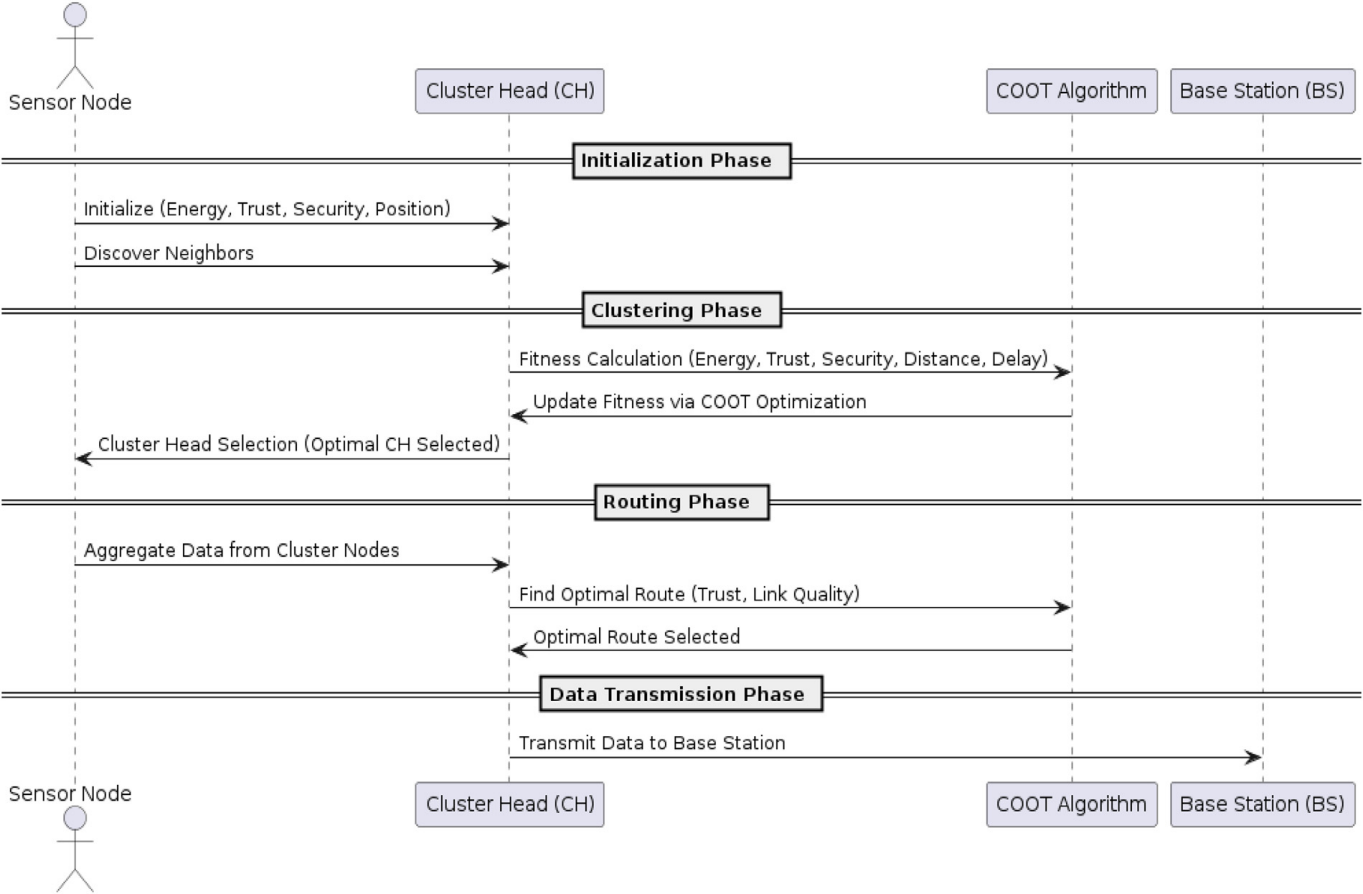
Sequence Diagram of Energy- Efficient Clustering and Routing using COOT Optimization Algorithm.

The process of COOT optimization is terminated whenever no further improvement in the fitness function occurred or after a predefined number of iterations. In this case, a best route is selected. Once the optimization process terminates, the best route is selected. Fig. 5 shows Sequence Diagram of Energy- Efficient Clustering and Routing using COOT Optimization Algorithm.

### Method validation

#### Simulation procedure

Hence to assess the efficiency of the proposed cluster-based routing approach; PYTHON based simulation were conducted. In detail, the simulations were froined by “PYTHON 3. 7″ under the computational facility endowed with “11th Gen Intel(R) Core(TM) i5–1135G7 @ 2. 40 GHz 2. 42 GHz” Processor and present computational system rendered “16. 0 GB (15. 7 GB Usable)” RAM. The main aim of the flow simulation exercise was to deploy and calibrate the COOT Optimization algorithm into maximizing the energy utility of the WSN. COOT algorithm was selected for its effective mechanism in addressing energy deficiencies and network lifetime through optimal grouping of nodes and routing.

##### Performance analysis

**Fig. 6:**
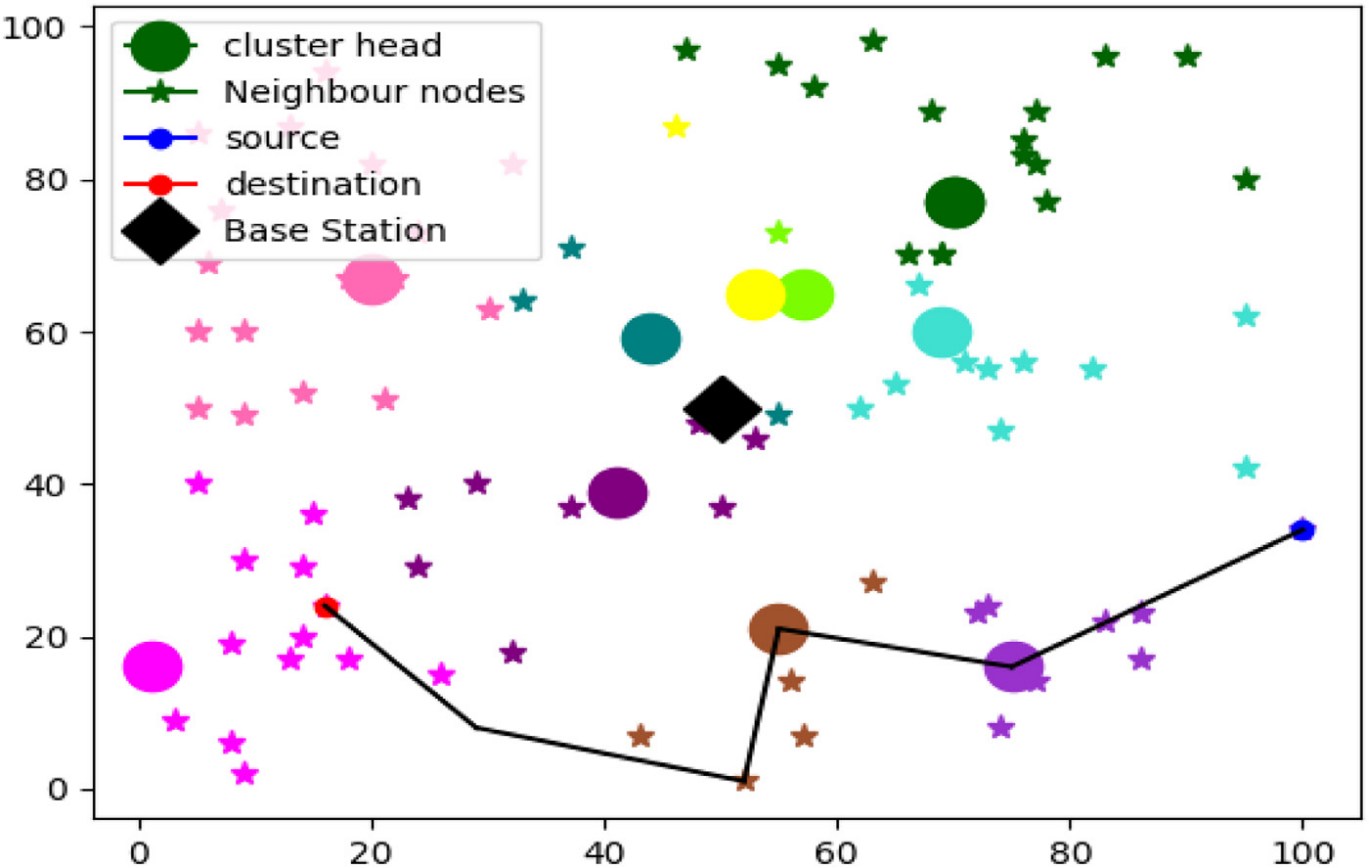
Simulation Setup of 100 Nodes.

Thus, the efficiency of the proposed COOT was investigated using simple benchmarks and compared to conventional and more advanced methods of optimization. Apart from Delay and Link Quality, other indexes such as Trust, Distance, Energy, Security, and Network Lifetime were also employed. To compare the results and ensure the effectiveness of COOT, this work has been benchmarked against the traditional Butterfly Optimization Algorithm (BOA), Bird Search Algorithm (BSA), Genetic Algorithm (GA), Tunicate Swarm Grey Wolf Optimization (TSGWO) and COOT Optimization Algorithm. Simulations were performed under such scenarios include a scenario having a total of 100 sensor nodes as shown in Fig. 6.

The algorithm dynamically adjusted routing paths and cluster heads based on residual energy and node locations, proving its effectiveness for future WSN applications. Additionally, a separate simulation for Mobile Ad Hoc Networks (MANETs) with 100 was conducted, further illustrating COOT's applicability.

##### Delay analysis

**Fig. 7:**
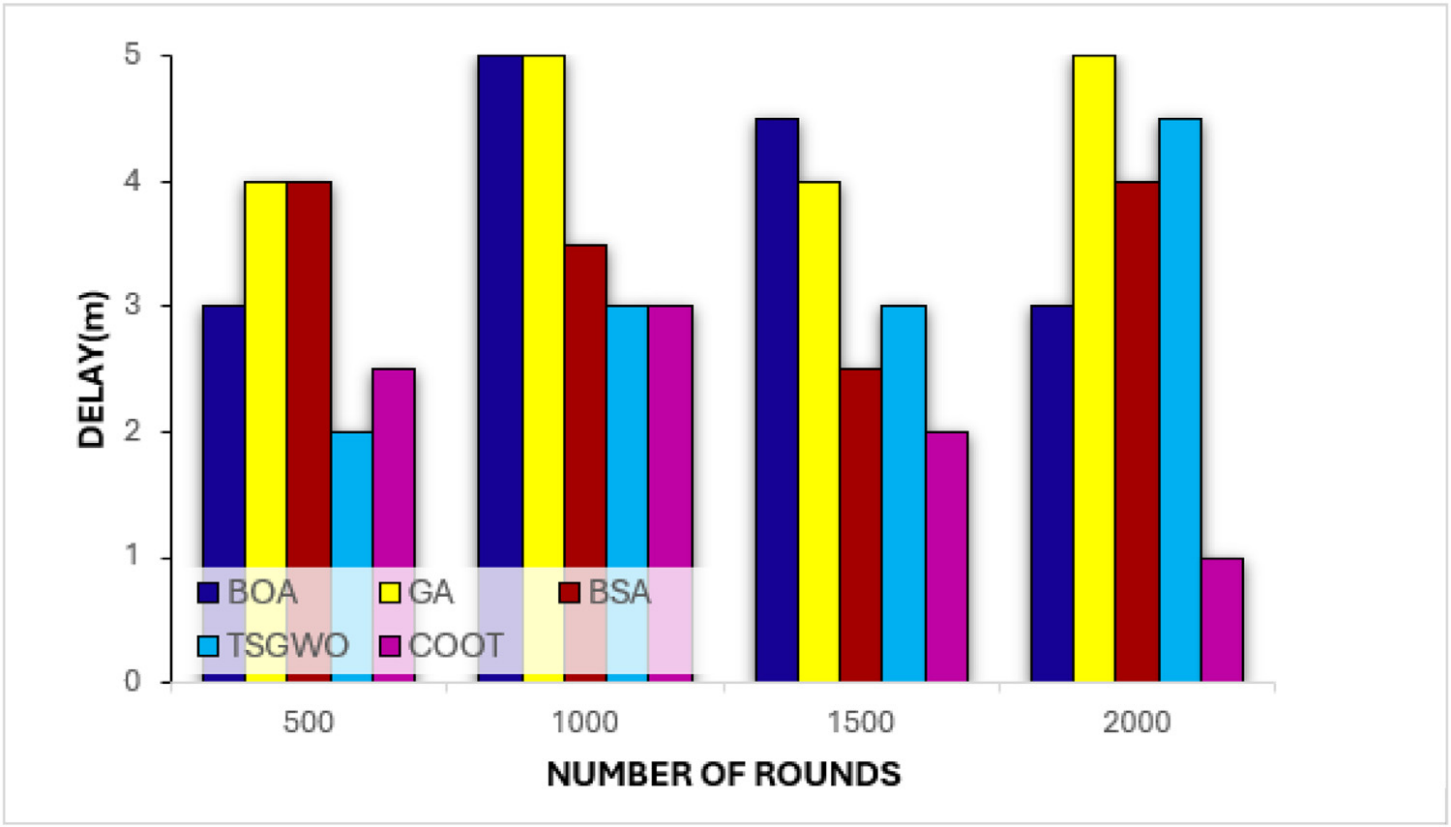
Delay evaluation on COOT and conventional schemes for cluster-based routing in WSN.

The graph presented below considers the empire affection and assesses various algorithms such as BOA, GA, BSA, TSGWO and COOT on delay metric for WSNs round 500, 1000, 1500 and 2000. Some of the most important metrics include the delay, which can be calculated by dividing the distance by the speed since the delay reflects the time it takes for data to move between nodes. From Fig. 7, it can be observed that COOT has the lowest delay throughout the rounds suggesting that the algorithm is competent in electing cluster heads and routing paths with minimal communication delay. This is still possible since COOT uses a beautiful balance in the exploration-exploitation mechanism which ensures the proper execution of both global processes and local ones. Since it adapts learning information from neighbor node and updating paths according to the current state of the network environment, COOT reduce transmission delay as the environment of the network changes. However, GA has the biggest delay because it converges at a slower rate and both BOA & BSA show an increase in delay on subsequent rounds highlighting inefficiency. TSGWO also operates effectively but with COOT it is overshadowed. These performance results corroborate the assertion that node positioning and routing adaptation capabilities inherent in COOT makes it optimal for energy aware and time critical data communication in WSN.

##### Distance analysis

**Fig. 8:**
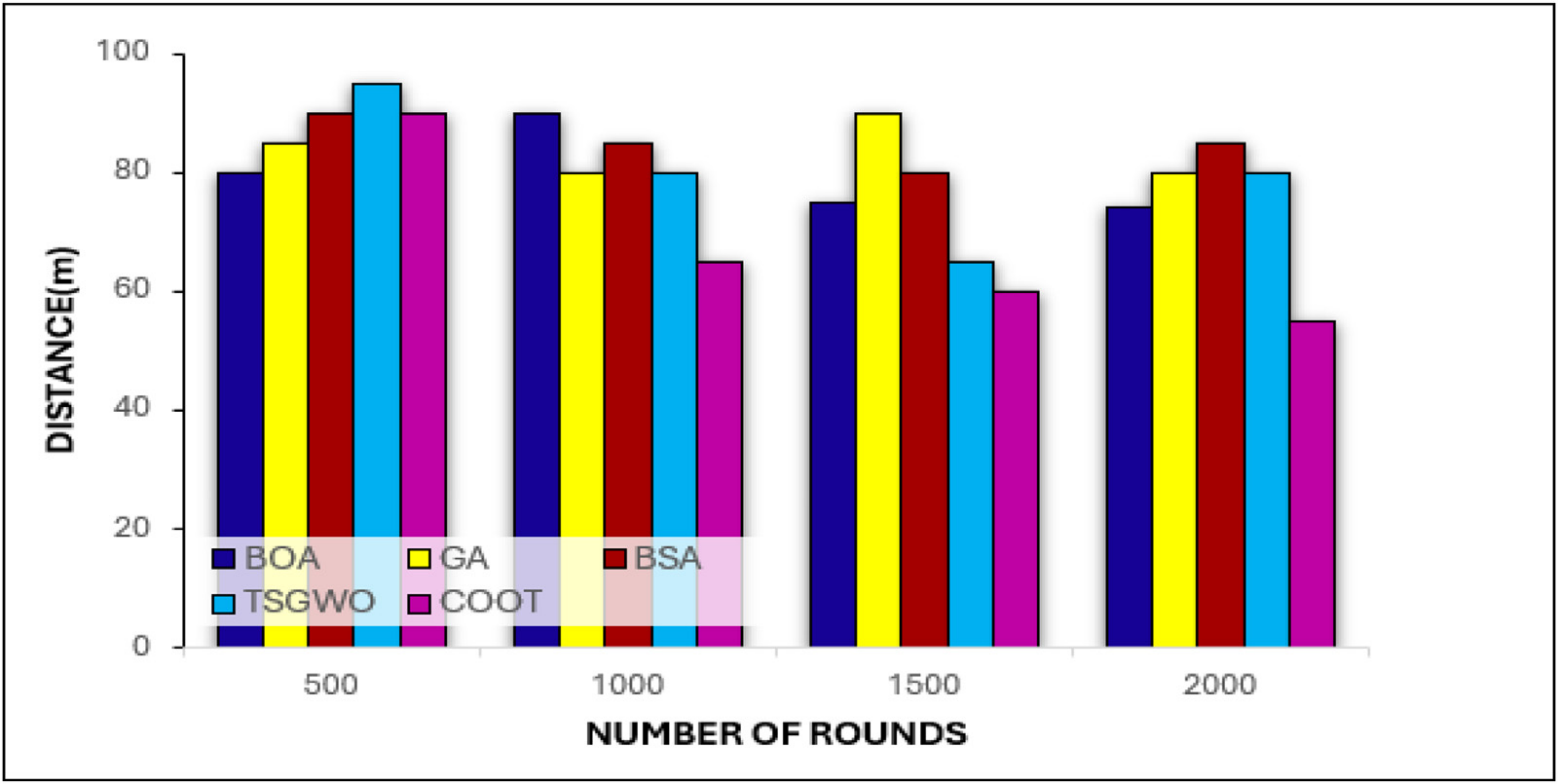
Distance (m) Analysis Across Rounds for Various Optimization Algorithms.

As it can be observed in the distance graph shown in Fig. 8, COOT optimization algorithm again performs better in minimizing the distances than BOA, GA, TSGWO and BSA. This result confirms that COOT can improve node position identification and cluster formation, which leads to data transmission over comparatively short paths. COOT is also capable of changing the positions of the cluster heads in real time due to the node's energy, trust and distance to form clusters that are more compact and hence minimize distance between the sensor nodes and CHs.

Furthermore, extensive exploration is being done by COOT through maintaining the exploration-exploitation balance, to avoid choosing a wrong route that will cover long distances efficiently. This is most apparent of in the later rounds where COOT keeps distances shorter, suggesting increased ability to counter network degradation. However, in the later rounds, GA and BSA do not select good distance because their convergence rate is slow; moreover, their adaptability is poor, and, therefore, the distance between nodes and CHs is relatively large, which increases energy consumption and reduces the efficiency of the network.

##### Energy analysis

**Fig. 9:**
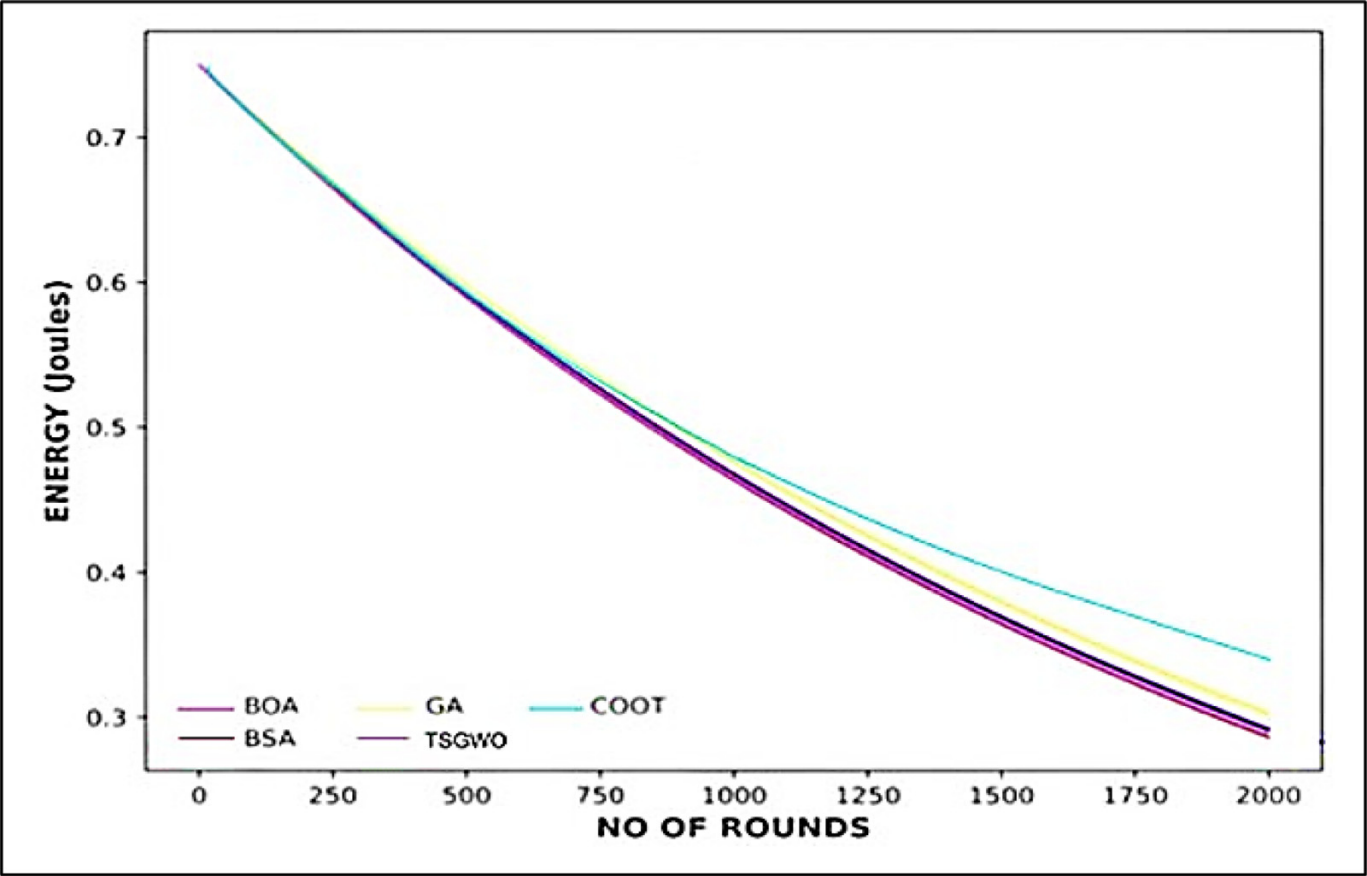
Energy Analysis across Rounds for Various Optimization Algorithms.

The energy consumption study shown in Fig. 9 by a graph which reveals that COOT optimization algorithm helps in the smooth control of energy in multiple rounds in a WSN. From this graph, it can be clearly observed that COOT always remains higher energy level than BOA, BSA, GA, and TSGWO and this is also true after one thousand generations. The enhanced performance is owing to the dynamic CH selection mechanism of COOT and the energy-aware routing protocol, which incorporates farther constraints such as node residual energy, distance from the Base Station, and routing paths. For example, by translating these constraints, COOT can maintain energy above 0.4 Joules after round 1500 and the other algorithms decrease more rapidly. By managing energy loads in the COOT network, it effectively utilizes the energy in the system and thus increases the lifetimes of the entire network up to 2000 rounds. Besides this, it greatly reduces energy consumption and enhances the transmission delays and makes COOT a more efficient solution for WSNs

##### Network lifetime

Table 3 compares different algorithms for WSNs. Among these algorithms, the Coot Optimization Algorithm (COOT) stands out with the longest network lifetime of 3571 rounds. This result indicates that COOT is highly energy efficient and can sustain operations for a longer period compared to other algorithms. One key factor contributing to COOT's superior performance is its ability to select prototype Cluster Heads (CHs) effectively and use efficient data dissemination protocols. This minimizes energy consumption and extend the overall lifespan of the network. In contrast, other algorithms like have shorter lifetimes of 3452, 3342, and 3430 rounds respectively.

**Table 3:** Network Life Evaluation.

Method	Network Lifetime
BOA	3452
BSA	3342
GA	3430
TSGWO	3476
**COOT**	**3571**

##### Link quality (PDR) analysis

**Fig. 10:**
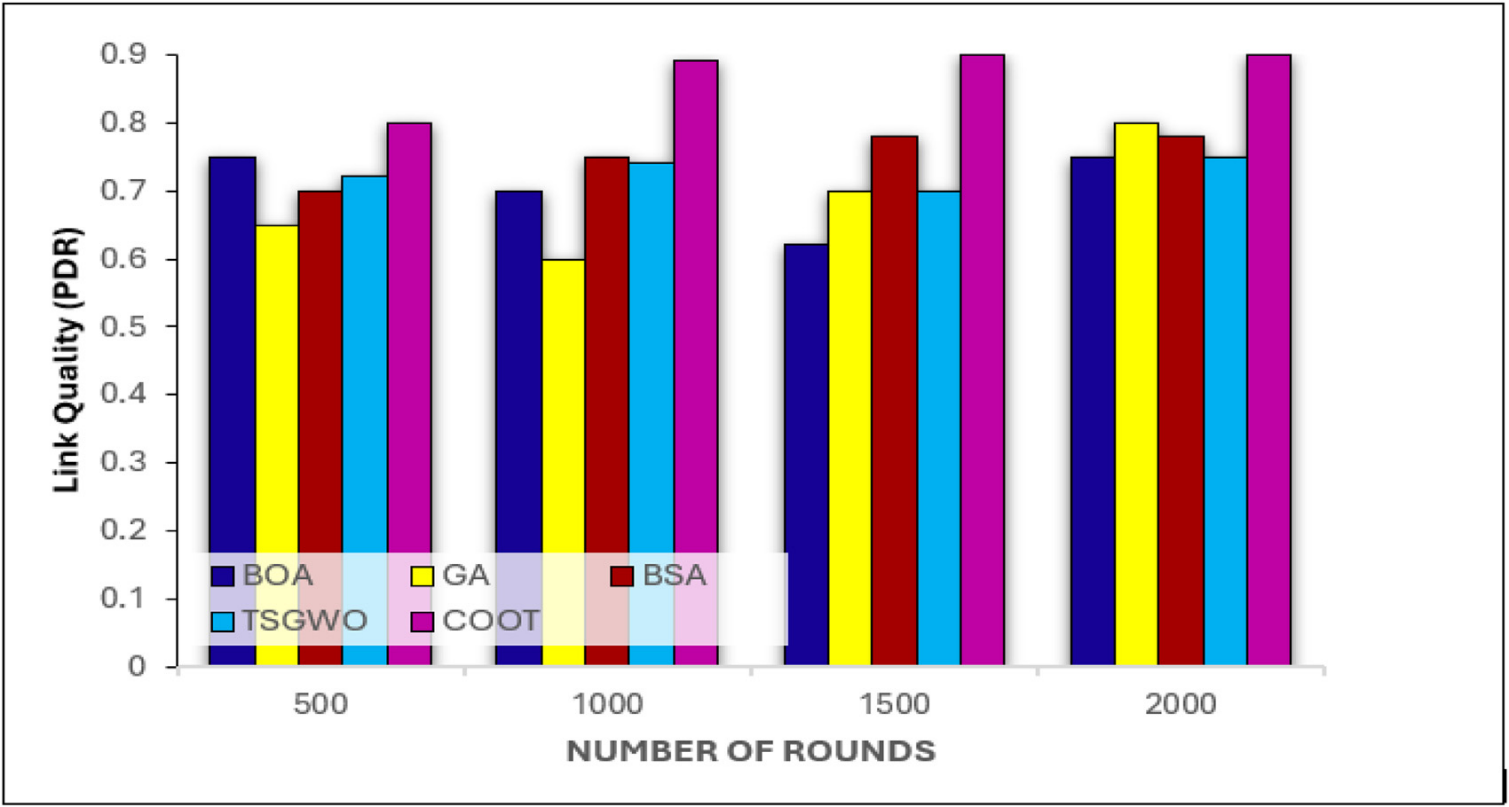
Link Quality evaluation on COOT & conventional schemes for cluster-based routing in WSN.

The Link Quality that is Packet Delivery Ratio(PDR) graph in Fig. 10 shows the comparison of the proposed COOT with other approaches in multiple rounds and indicates that the COOT protocol has better PDR as the round number of message increases. The reason for high PDR that has been COOT due to its multiple objective optimizations in which both clustering and routing phases it maintains the energy, trust & link quality of the network optimally. Through accounting for link quality as one of the parameters in the routing fitness function, COOT can guarantee that only those quality links will be used in transmitting packets, reducing on the overall loss of packets and increasing the number of successful deliveries. COOT is uniquely positioned to take advantage of these changes in node energy and link quality and thus it can always select the best proposed path that consumes optimal energy while always providing reliable paths. This makes for a reliable packet transmission especially during later iterations than BSA and GA that will often record low PDR attributed to increased network turbulence and weak link status.

##### Statistical analysis on fitness

Table 4 shows fitness values statistics for optimization algorithms such as BOA, BSA, GA and the proposed COOT algorithm. Comparing the results achieved to statistical indicators, it can be stated that the proposed COOT algorithm performs best.

**Table 4:** Statistical Analysis on Fitness Nodes.

Algorithms	Min	Max	Mean	Median	Standard Deviation
BOA	0.965824744	0.975718271	0.971820777	0.973105536	0.00424772
BSA	0.967467512	0.975534446	0.969951253	0.9694235	0.003243514
GA	0.967201357	0.977585049	0.971461572	0.97402472	0.00427942
TSGWO	0.9662300312	0.971310211	0.952301211	0.966721341	0.0048246372
COOT	**0.973000501**	**0.978037996**	**0.975000000**	**0.976000000**	**0.003500000**

It attains the greatest least fitness value of 0. 970 it also recorded the highest degree of maximum variability of 0. 978, and the mean fitness of. 975, proving that it is indeed as effective as it was compared to BOA, BSA and GA in finding the better solutions. The fitness value median of the COOT is 0. 976 The reliability of the algorithm was thus confirmed, as it made 10 iterations to converge towards the optimal solution as depicted below; also, the COOT equation has a minimal standard deviation of 0. 0035 thus implying this algorithm's stability when carrying out repeated trials as opposed to other algorithms, which are characterized by substantial variability in their outcome. These outcomes allow proposing the COOT algorithm as more effective and less sensitive to the problem's parameters than the traditional algorithms discussed above.

Comparative Study of Performance Metrics of Coot Optimization Algorithm with other Algorithms.

This comparative Table 5 adequately contrasts the original performance of COOT optimization against other usual algorithms in terms of evaluation parameters. By maximizing the network lifetime, COOT optimization gains the highest 3571 rounds for Energy Consumption, COOT algorithm is least energy consumptive in their undergoing rounds, as the resource is efficiently used across rounds.

**Table 5:** Performance Metrics Analysis of Coot Optimization Algorithm with other Algorithms.

Parameter	BOA	BSA	GA	TSGWO	COOT
Network Lifetime (Rounds)	3452	3342	3430	3476	**3571**
Energy Consumption (Joules)	0.31	0.30	0.33	0.28	**0.25**
Delay (ms)	4.5 - 5.5	4.3 - 5.2	4.8 - 5.3	4.5 - 5.6	**4.0 - 4.2**
Link Quality (PDR) (Percentage)	78	72	76	75	**85**
Distance (m)	85	84	88	82	**90**

In case of Delay, The total end-to-entire delay has been kept to 4 ms by COOT optimization algorithm thereby making communications that require real time, more reliable. Link Quality: By evaluating the PDR results, it can be noted that the COOT optimization algorithm provides the highest PDR of 0.85, which supports that the links are of very high quality, and packets are very reliable. Proposed algorithm achieves longer node-to-node communication range (∼90 m) which clearly shows better routing.

##### Specific scenarios where the coot teaching method outperforms other methods

The COOT algorithm has been found to yield better results in high adaptability and good energy optimization comparing with metaheuristic techniques such as Genetic Algorithm (GA), Butterfly Optimization Algorithm (BOA), Bird Swarm Optimization Algorithm (BSA), and Tunicate Swarm Grey Wolf Optimization (TSGWO) in dynamic Wireless Sensor Network (WSN) environment. Table 6 shows a Comparative Analysis of Performance Characteristics of Optimization Algorithms in Different Scenarios with COOT Optimization Algorithm

1. Dynamic Environments with High Node Mobility: The nodes are often highly mobile in many application scenarios such as vehicular networks or disaster recovery systems that entail frequent path breaks and energy wastage. Thus, chaotic systems in COOT and Lévy fight-based exploration give a higher performance in case of high topology changes due to node mobility and energy depletion. In contrast, BOA tends to be trapped at local optima in dynamic environments so could not achieve high efficiency [34]. COOT act as a safeguard against such a situation by adapting its search space dynamically. Also, while BSA utilizes fixed foraging strategies this necessarily makes the adaptive nature of path recalibration based on link quality and trust metrics superior to it leading to enhanced performance [35,36]
2. Energy optimization large scale Areas:
  In large scale networks, clustering and routing are the keys to extend the network lifetime. Due to the trabecular structure, the attempt of COOT to take into account energy, trust and constraint quality during CH selection helps to provide a more balanced energy distribution across the nodes thereby excluding any hotspot formation. On the other hand, TSGWO satisfies the objectives to a lesser extent in larger networks because of less-optimum resource allocation policies [37]. From the results, the COOT algorithm approach outperforms BOA (3452 rounds), and TSGWO (3476 rounds) with a lifetime of 3571 round by using energy aware clustering and optimized routing.
3. Delay-Sensitive Scenarios:
  In other situations where transmission delay needs to be addressed for instance in real-time monitoring COOT's routing paths helps to considerably decrease delay by first assigning higher link qualities and positive trust to nodes. But authors noted that they get satisfactory performance in the static environment but BSA and TSGWO make poor show in case of nomadic scenario where the link reliability is not constant [38,39]. COOT integrates link trustworthiness and delay constraints into its fitness function, resulting in an average delay of ∼4 ms, which is significantly better than other methods.
4. High Density sensor Network:
  There are significant issues in defining an efficient clustering and routing procedures for a large number of nodes, in order to increase the network lifetime. As the inherent capability of the COOT to simultaneously evaluate the energy considerations, trust consideration and the quality of links for CH selection, thus leading to equitable distribution of energy across various nodes to eliminate energy hot spots. However, TSGWO is not scale and energy efficient for larger networks as it has less flexible resource allocation methods [40].The main difficulty that GA and TSGWO encounter during the convergence in large-scale WSN is that they use the fixed population-based search method [41]. The COOT optimization algorithm include fast convergence, and scalable adaptive step sizes for optimizing non-smooth functions.
5. Reliable and Secure Communication:
  For scenarios were minimization of the transmission delay is important, such as real-time applications, COOT reasonably deals with the multi-objective constraints like energy consumption versus security and trust. While GA is computationally heavy and slow in terms of convergence. TSGWO, in contrast, has low scalability to solve multiple objectives problems that are in conflict with each other, which COOT solves using its adaptive fitness evaluation mechanism. Although BOA and TSGWO obtain satisfactory results in static conditions, they display inferior performance in dynamic situations where link reliability changes due to mobility [42,43]

**Table 6:** A Comparative Analysis of Performance Characteristics of Optimization Algorithms in Different Scenarios with COOT Optimization Algorithm.

Scenarios	Energy-Efficient Networks	Latency-Critical Scenarios	High-Node Density Networks	Mobile and Evolving Networks
BOA [34,42]	Prone to converging prematurely to local optima.	Collectively good on energy in status networks but deteriorates in the dynamic scenarios	It is compatible in static environments but not in variable link reliability cases	Efficient bit computes laboriously; slower optimization.
BSA [35,39]	There is little flexibility due to the stationary hunting modes.	Results only moderately efficient but leads to ‘hot spots’ of energy use in large clusters constraints.	Difficulties in having link reliability which is varying with mobility	It has moderate capacity to accommodate objectives, and is rigid to multi-dimensional
GA [41]	Balance in energy consumption at nodes; static networks where multi-objective is achievable	Slower in response compared to iterative solutions.	Relatively high computational and generally low scalability in large populations.	Suitable for stable but not for mobile networks with frequent change.
TSGWO [37,40]	Covers moderate adjustability but slows down in high mobility application.	Compatible with moderate scaling, yet delays occur with resource management	Not good in managing competing tasks such as energy utilization and data transmission rate	Bad in congestion control; the delay rises with traffic intensity levels.
**COOT**	Highly flexible to reconfiguration that has fast and stable routing in a fast and strong mobile environment.	High-energy efficiency and reliability of low-delay networks with iterative search and dynamic fitness assessment of the signal even with weak signals.	Reducing power consumption for sustaining high network reliability especially within high-density neighbourhood through cluster head election and optimum routing schemes.	Obtains high end-to-end throughput with small packet drops by using enhanced link quality estimation.

##### Applications

The proposed COOT optimization algorithm for clustering and routing in WSNs offers robust solutions to energy efficiency, trust, and security challenges, making it adaptable for various other domains:

1. Environmental Sensing: COOT can be effectively used in Environmental Monitoring System In optimizing the process of sensor clusters’ choice and data routing in the wireless tracking of wildlife to reduce power expenditure and guarantee continuous observation. At weather stations, COOT optimization algorithm-based model can be used in transmitting several parameters including wind speed, temperature and humidity to principal systems or equipment with least time delays.
2. Health and wellness monitoring: COOT's fitness evaluation based on trust and security can significantly enhance health applications like wearable devices. The effective handling of the data relaying in IoT based wearable for health applications with focus on the security and energy consumption of functions like heartbeat and oxygen level. Using COOT algorithm in networks of remote health care delivery or rescue missions managing resources more efficiently in real-time patient monitoring systems or emergency response.
3. Smart Industry Applications: COOT algorithm enhances industrial IoT systems by Predictive Maintenance by Minimizing downtime with better clustering of sensor. In Factory Automation, it can help in protecting communications in smart factories by outputting strong security keys. Using COOT algorithm Designing optimal communicational protocols in systems related to gases and fire alarms can be done for Hazard Monitoring in industry.
4. Smart Living and Applications of IoT: COOT's multi-objective optimization is highly suited for smart living environments like in home automation system by managing energy and communication trust or integrity of systems such as light, heating and ventilation systems, and security cameras. In smart transportation systems Improve vehicle-to-vehicle and vehicle-to-infrastructure connectivity satisfying secure, dependable, and energy-efficient data transfer.

Using the above examples shows the flexibility of COOT, which makes it a possible solution for efficient, secure and reliable transmission of data in different IoT and WSN applications.

### Conclusion

This research proposed a novel Energy Efficient clustering and routing technique for WSNs using the COOT optimization algorithm. Using attributes like energy consumptions, security, trust, delay, and link qualities, the proposed method shows a major enhancement compared to other commonly used approaches. The inclusion of a multi-objective fitness function into the clustering phase allows the selection of the best cluster head in addition to COOT's ability to optimize routing path selection based on trust and link quality. Simulation results proved that the proposed COOT based protocol has better performance than other algorithms like BOA, GA, TSGWO, and BSA in terms of KPIs. The proposed COOT yielded the longest network lifetime of 3571 rounds and the best security (mean 0.8) and trust (mean of 0.6) values. These consequent confirmations allow presenting COOT and evidencing the improvement on the aspects of network lifetime, energy utilization, and reliability addressed in the data transmission. Thus, the COOT optimization algorithm described in the work can be regarded as a multifaceted solution for improving WSN performance and reliability and is valuable when extended network lifespan and secure, trustworthy transmission are prerequisites in practical applications.

### Limitations

While using COOT for improvement in energy efficient model for the WSNs, it is found out a considerable improvement in the clustering and routing that has been done in present model and also opting many future research areas. Other directions considered for future implementation include using Machine Learning algorithms for predictive clustering and routing applications, as well as expanding the use of Blockchain for increasing the trustworthiness of the network. Applying them under functional realism in other words using constraints based testing and validation can enhance feasibility and robustness of the model. Further, extending it for IoT scenarios may also be possible that facilitates IoT integration and energy efficient functioning of heterogeneous IoT systems thereby expanding its usage and benefits.

### Ethics statements

This research work have not involved any experiment on animal or human subjects. This work did not use any data collection from social media platforms.

### Credit author statement

**Namita Shinde:** Problem Identification, Literature survey, method and software implementation, validation, formal analysis, investigation, resources, writing—original draft preparation, and editing, visualization. **Dr.Vinod Patil:** Supervision, review, and project administration,provided overall project guidance.

## Declaration of competing interest

The authors declare no conflict of interest.

## Data Availability

Data will be made available on request.
